# Effect of hydrophobically modified PEO polymers (PEO-dodecyl) on oil/water microemulsion properties: *in vitro* and *in silico* investigations

**DOI:** 10.1039/d0ra09804c

**Published:** 2021-02-10

**Authors:** M. Khatouri, R. Ahfir, M. Lemaalam, S. El Khaoui, A. Derouiche, M. Filali

**Affiliations:** Laboratoire de Physique Appliquée, Informatique et Statistique (LPAIS), Faculty of Sciences Dhar El Mahraz, Sidi Mohamed Ben Abdellah University BP 1796 Fes Atlas Morocco; Laboratoire de Physique des Polymères et Phénomènes Critiques Sciences Faculty Ben M'Sik, Hassan II University P.O. Box 7955 Casablanca Morocco mohammedlemaalem@gmail.com lemaalemmohammed@gmail.com

## Abstract

Microemulsions are excellent systems for transdermal delivery of multifunctional drugs because they have the potential to improve drug absorption/permeation and handling limitations. Biocompatible polymers are used as a coating of microemulsions to avoid the interactions that can occur between the microemulsions and the skin. Thus, they protect and lubricate these transporter nanovesicles. In this paper, we studied decane/water microemulsions covered with hydrophobically modified PEO polymer (PEO-m). To reveal the effect of hydrophobically modified PEO (PEO-m) polymer on the shape, the micro-arrangement and the dynamics of the microemulsions, we used an integrated strategy combining Molecular Dynamics simulation (MD), Small-Angle Neutron Scattering experiments (SANS), and the Ornstein–Zernike integral equations with the Hypernetted Chain (HNC) closure relation. We determined the microemulsion shape *in vitro* using the renormalized intensities spectra from SANS experiments. We discussed the micro arrangements of microemulsions, *in vitro* and *in silico*, employing the pair correlation function *g*(*r*) and the structure factor *S*(*q*), obtained from the three approaches with good agreement. Thus, we used the validated MD simulations to calculate the microemulsion's dynamics properties that we discussed using the mean-squared displacement (MSD) and the diffusion coefficients. We found that the presence of moderate quantities of PEO-m, from 4 to 12 PEO-m per microemulsion, does not influence the microemulsion shape, increases the stability of the microemulsion, and slightly decrease the dynamics. Our *in vitro* and *in silico* results suggest that polymer incorporation, which has interesting *in vivo* implications, has no disadvantageous effects on the microemulsion properties.

## Abstract 2

In this work, we study the effect of grafted PEO-dodecyl co-polymers on the decane/water microemulsions properties. For this purpose, we combined the MD simulations, the OZ integral equations resolved using the HNC closure, and SANS experiments.

## Graphical abstract

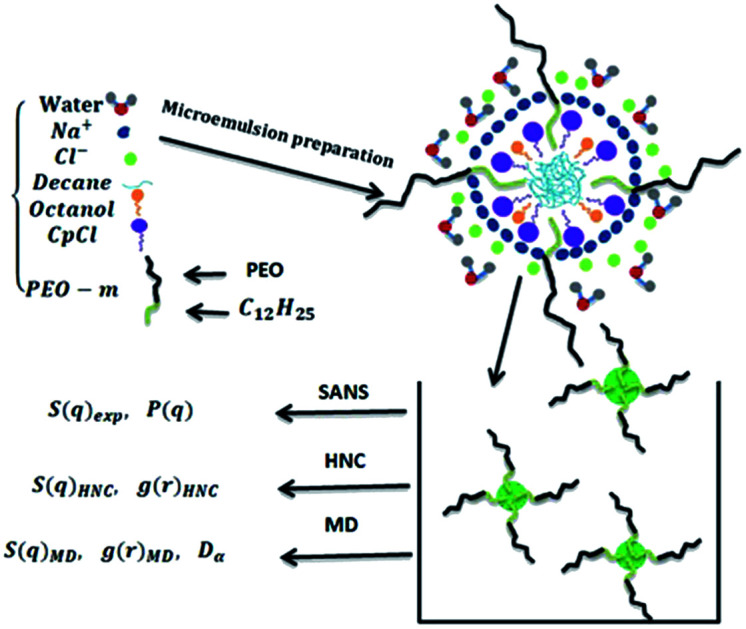

## 1 Introduction

Microemulsions are nano-transporters formed by self-assembly approaches, similar to that of biological cell membranes. In recent years, bioinspired microemulsions have attracted a lot of attention because they promise great potential for drug delivery and pharmacological prospects.^1–4^ Like liposomes, microemulsions have also hydrophobic and hydrophilic compartmentalized micro-domains, in which hydrophilic and lipophilic drug molecules could be encapsulated and stabilized.^5–7^ In addition, microemulsions are the subject of many potential applications in detergents,^8^ lubrication,^9^ phytosanitary,^10^ cosmetics,^11^ formulation of paints,^12^ and advanced petrol extraction.^13^ This growing interest in microemulsions also results from a better understanding of their physicochemical properties.^14–17^ The interfacial film formed by the surfactant molecules is characterized by its tension, its rigidity, and its spontaneous curvature.^18–21^ Their relative importance depends on the stresses undergone by the film which determines the physical properties of microemulsions such as their phase behavior, their stability, their structure, and their solubilization capacity.^22–25^

Although there are still few products on the market, microemulsions exhibit remarkable properties, in particular an ultra-low water/oil interfacial tension^26,27^ and a high solubilizing power both towards hydrophilic and lipophilic compounds.^28,29^ Compared to a conventional emulsion, a microemulsion is a better vector for the active agents because it consists of nanodroplets that allow better penetration of these active agents.^30–32^ It also has the advantage of being prepared cold,^33–35^ which on the one hand reduces energy consumption during its preparation, and on the other hand, it uses heat-sensitive active ingredients. Thus, in comparison with micellar solutions, microemulsions can solubilize a greater quantity of active agents due to their greater stability partly due to the presence of a greater quantity of surfactant, which can, on the other hand, constitute an obstacle to their use for reasons of cost and/or toxicity. Nevertheless, microemulsions improve the rate and degree of absorption of lipophilic drugs. Advantages offered by microemulsions include improved drug solubility, improved bioavailability, environmental protection of the drug, ease of manufacture, and long shelf life.^36–41^

Several works have performed *in vitro* and *in silico*, to study the influence of polymers incorporation on the microemulsions system. In this context, Soheil Sharifi *et al.* used the photon correlation spectroscopy technique to study in detail the effect of polyethylene glycol (PEG) on the droplet dynamics of water droplets in the inverse microemulsion of sodium bis-(2-ethylhexyl)sulphosuccinate (AOT), they also used the small-angle X-ray scattering technique to study the structure and to control the size and polydispersity of the mixing system (AOT/PEG microemulsion). They observed a single relaxation when adding PEG to the microemulsion for the lower molecular weights. In contrast, for the high molecular weights, they observed a double relaxation process. As the concentration of the polymer increased, the diffusion coefficient decreased for the polymer length scale of *M*_w_ = 285. But for the two higher molecular weights (*M*_w_ = 2200 and 6000), the diffusion decreases with increasing PEG content. Besides, SAXS experiments show that by increasing the concentration and length scale of PEG, the microemulsions size remains constant, and the polydispersity increases.^42^

P. M. D. Molina *et al.* used the small-angle X-ray scattering (SAXS) and Dynamic Light Scattering (DLS) and Fluorescence Correlation Spectroscopy (FCS) methods to study the structure, dynamics and rheology of a diluted O/W microemulsion with the addition of increasing quantities of a telechelic polymer on the microemulsion. They showed that the size of the microemulsions is not affected by the addition of polymer but induces attractive interactions at low concentrations and repulsive interactions at high concentrations of the polymer. They have also shown that at higher polymer concentration, the formation of the network leads to an additional slow relaxation mode in the DLS which can be related to rheological behaviour, while the self-diffusion observed in the FCS reaches a lower plateau value.^43^

Argyrios Karatrantos *et al.* used molecular dynamics simulation to study the dynamic properties of nanoparticles and polymers in nanocomposites containing spherical nanoparticles. They showed that small nanoparticles diffuse much faster than predicted by the Stokes–Einstein relationship in the dilute regime. Also, they noticed that when the nanoparticle loading is higher, the diffusivity of the nanoparticles decreases due to the nanoparticle–polymer interface. Besides, the increase in the radius of the nanoparticle slows down their diffusion.^44^ Valerio Sorichetti *et al.* used molecular dynamics simulation to study a semi-diluted non-entangled polymer solution containing nanoparticles, whose size is less than the polymers radius of gyration. They revealed that the diffusion coefficient of polymer chains and nanoparticles decreases if the volume fraction of the nanoparticles is increased.^45^

Hoffmann *et al.* used the neutron spin-echo (NSE) technique and dynamic light scattering (DLS) to study the dynamic properties of oil/water microemulsions bridged with telechelic polymers of different arms numbers. From the NSE results, they showed that the linear polymer is more effective in stopping the movement of individual droplets of ME and from the DLS results observed that polymer bridging has a weak influence on the local dynamics, even though the addition of polymer leads to an increase in viscosity by several orders of magnitude.^46^ Pooja Nath *et al.* used fluorescence correlation spectroscopy (FCS) and single-particle tracking (SPT) experiments to study probes of nanoparticles dispersed in simple isotropic liquids and polymer solutions. They have shown that in simple fluids and for polymer solutions in which the particle diameter is much larger than the radius of gyration *d* ≫ *R*_g_, the long-term particle dynamics are driven by random walk statistics, with the overall zero shear viscosity of the polymer solution that determines the frictional resistance to particle motion. On the other hand, in polymer solutions with a particle diameter less than the radius of gyration, the polymer molecules in solution exert non-continuous resistance to particle movement, and the nanoparticle probes appear to interact hydrodynamically only with a local fluid medium with an efficient drag comparable to that of a polymer melt to the nanoparticle probes.^47^

**Fig. 1:**
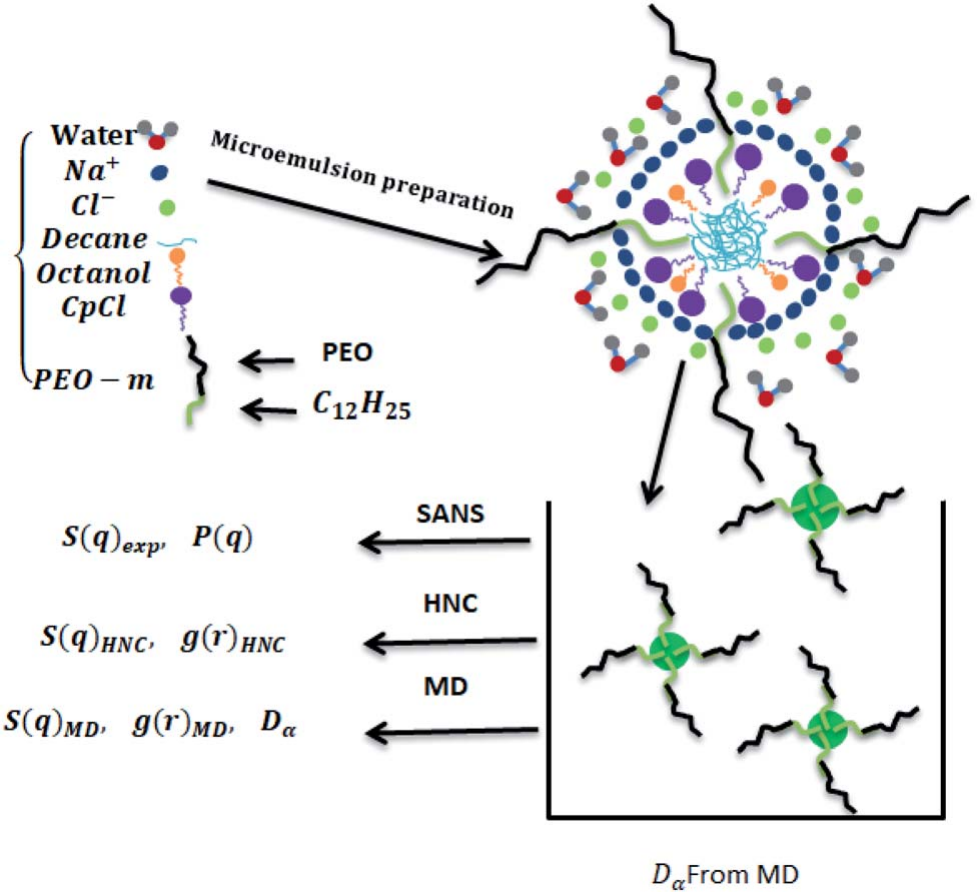
Schematic representation of the measurements performed in this work.

The decane/water microemulsions are prepared with incorporated biocompatible polymers, as depicted in Fig. 1, which maintain its stability in the intravenous environment. The used polymers consist of poly(ethylene oxide) (PEO), a common polymer in drug delivery,^48,49^ with added dodecyl hydrophobic chain (C_12_H_25_) to one of its ends, that we note PEO-m. These polymers prolong the blood circulation time of the microemulsions, just like liposomes.^50–52^ Thus, the accumulation of drugs could be significantly improved, within the target diseased sites, with the presence of polymers. Thus, the covered nanovesicles enhance the effect of permeation and retention.^53,54^

We recall that for the microemulsions/polymers mixed system, the polymers can introduce steric or attractive interaction types,^55^ depending on three main factors.

First, they depend on the quality of the solvent conditions. The steric type interactions are present for the good-solvent case. These interactions are attributed to the osmotic interactions between the polymers segments adsorbed on the microemulsions surfaces.^56^ In contrast, for the bad-solvent condition, the repulsive interaction weakens. Thus, the steric repulsion can not overcompensate the van der Waals attraction. Consequently, the microemulsions aggregate.^57^

Second, the bridging effect, where polymer chain adsorbs on two or more microemulsions simultaneously. Thus, the polymer causing a strong attraction interaction, which leads to flocculation even under good-solvent conditions.^58^

Third, the interactions between the surfactant molecules and the added polymers. These interactions are controlled by van der Waals forces, the dispersion forces, the hydrophilic effect, and electrostatic interactions.^59^ Accordingly, these interactions lead to adsorbed, or non adsorbed, polymers to the microemulsions surface. In the case of adsorption, the polymers form a shield around the microemulsions. Thus, lead to the steric effect. On the opposite, non-adsorbed polymers can lead to an attractive interaction between the microemulsions.

In this study, we add a co-polymer that we note PEO-m to decane/water microemulsions. The PEO-m consists of a hydrophilic polyethylene oxide chain (PEO) and a hydrophilic alkane chain. The microemulsions are stabilized by cetylpyridinium chloride surfactant and octanol cosurfactant. In this case, the hydrophobic alkane chain belongs to the PEO-m is adsorbed to the microemulsion surfactants. Also, the PEO-m is considered in good solvent condition and has no bridging effect because of its hydrophilic end. Thus, the PEO-m introduces a steric-type repulsive interaction.

In the following, we describe the preparation of the studied samples, and we investigate the effect of the added polymer PEO-m on the shape and size of the microemulsions using SANS experiments. Also, we examine this effect on microemulsions micro-distribution using SANS, HNC numerical method, and MD simulations. Furthermore, we investigate the microemulsions dynamics in the presence of adsorbed PEO-m polymers using MD simulations.

## 2 Materials and methods

### 2.1 Materials

Cetylpyridinium chloride CpCl, decane, octanol, and the poly(ethylene oxide) (PEO) are purchased from Fluka. CpCl has purified by successive recrystallizations in water and acetone. The PEO has hydrophobically modified, by adding dodecyl hydrophobic chain to one of its ends and purified in the laboratory, according to the method described in the ref. 60 and 61. The molecular weight of the starting materials, we present in Table 1, is determined by using size exclusion chromatography (SEC).^62,63^ The hydrophobically modified PEO contains an isocyanate group between the alkyl chain and the ethylene oxide chain. We assume that this isocyanate group belongs to the hydrophilic part of the copolymer. We note that the PEO-m is modified at one extremity only.

**Table 1:** Molecular masses and densities of the different molecules we used in the preparation of the covered microemulsions

Components	Molar mass (dalton)	Density (g (cm)^−3^)
H_2_O	18	1
D_2_O	20	1.105
[H_3_C(CH_2_)_15_]C_5_H_5_N^+^Cl^−^ (CpCl)	339.5	1.656
[H_3_C(CH_2_)_7_]OH (octanol)	130	1.8
[H_3_C(CH_2_)_8_CH_3_] (decane)	142	0.73
[CH_3_(CH_2_)_11_NHCO(OCH_2_CH_2_)_227_O(CO)NH] (PEO-m)	5200	1.2

### 2.2 Preparation of the decane/water microemulsion with the presence of PEO-m polymers

The oil/water microemulsions studied in this work are formed by cetylpyridinium chloride (CpCl) as a cationic surfactant, *n*-octanol as co-surfactant, decane as oil, and saline water (0.2 mol l^−1^) as a solvent. The mass ratio of oil to the surfactant film was 0.62. The latter represents the oil quantity present in a microemulsion. The mass ratio between surfactant and co-surfactant is 0.25. The latter controls the curvature of the hydrophilic–hydrophobic interface. We assume that the volume fraction *Φ* remains constant when adding modified polymers to the microemulsions. To characterize a microemulsion with grafted modified polymers, we introduce a parameter that we note m. The latter indicates the dodecyl hydrophobic chain added to the PEO end. Thus, PEO-m indicates the modified PEO. We prepared eight samples: a diluted microemulsions system (*Φ* = 6.98%) and a concentrated one (*Φ* = 25.6%). In each case, four different numbers of PEO-m per microemulsion are added (0, 4, 8, and 12).

All samples are prepared by weight. Their overall composition is determined to obtain a constant volume fraction of the microemulsions while increasing, progressively, the number of adsorbed PEO-m by replacing a small number of surfactant molecules with the appropriate amount of PEO-m. We characterized the solutions by the volume fraction and the number of added PEO-m chains per microemulsions (*N*_p_). To calculate *N*_p_, we assume that the microemulsion radius does not change with increasing substitution of the surfactant by the PEO-m copolymers. It is important to stress here that, with the present choice of PEO-m, a sample prepared with the same value of *N*_p_ contains the same number of PEO-m chains per microemulsion. The prepared samples are homogenized and kept in a thermostated water bath *T* = 20 °C for several days. When we observe a phase separation, the prepared samples are homogenized again and set back to rest for a couple of days to confirm these observations.

### 2.3 Small angle neutron scattering (SANS) measurements

The small-angle neutron scattering experiments have been performed at LLB, Saclay, on the PACE spectrometer at the room temperature *T* = 25 °C. The configurations used were: 1.5 m at 6 Å and 4.68 m at 13 Å. Thus, the corresponding accessible wave vectors were in the range 0.004 Å^−1^ < *q* < 0.16 Å^−1^. The scattering data are scaled to an absolute scale using water as standard. Thus, the intensities obtained in the absolute units (cm^−1^), with accuracy better than 10%.

In the framework of SANS experiments, the scattered intensity expressed as:

1 *I*(*q*) = *Φv*(Δ*ρ*)^2^*P*(*q*)*S*(*q*)

where *q* (Å^−1^) depict the diffusion vector, *Φ* stand of the volume fraction, *v* (cm^3^) represent the dry volume of the aggregates, and Δ*ρ* = 6.83 × 10^10^ cm^−2^ is a parameter representing the difference between the diffusion length density of the nanoparticles and the solvent, corresponding to the contrast of the sphere. *P*(*q*) is the form factor of the colloidal aggregates, where *P*(*q* → 0) = 1. *S*(*q*) is the structure factor which provides information on the microemulsions arrangement and thus on their interaction potential.

### 2.4 Ornstein–Zernike integral-equation method

The Ornstein–Zernike equation (OZE) is as follows:^64^2
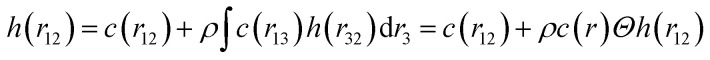
where *h*(*r*) is the total correlation function, *c*(*r*) is the direct correlation function and *Θ* symbolizes the convolution product. The *h*(*r*) between two particles 1 and 2 is related to the pair distribution function *g*(*r*) by the relation, *h*(*r*) = *g*(*r*) − 1. Thus, *h*(*r*) is the sum of a direct correlation *c*(*r*) and all indirect correlations between 1 and 2 implicating an increasing number of intermediate particles 3.

The OZE contains two unknown functions, namely, *h*(*r*) and *c*(*r*). Consequently, we must introduce another relation to close the system. From the literature, there are different relations called integral equations derived from simple liquid statistical mechanics, which admit analytical or numerical solutions and which are more or less approximate. In this work, we have used the so-called hypernetted-chain equation (HNC):^65^3
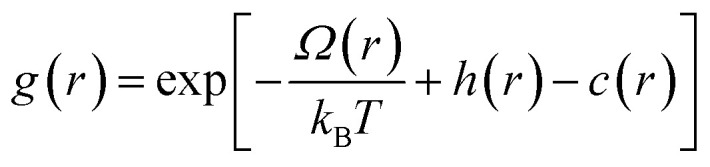
where *Ω*(*r*) the mean interaction potential. Then, this non-linear integral equation is resolved numerically.

Many structural and thermodynamic quantities are depending on the pair distribution function. Thus, the knowledge of the pair correlation function, also called radial distribution function *g*(*r*), along with the interaction potential *V*(*r*), in the case of an isotropic system, allows the calculation of the internal energy *U*,^64,65^4
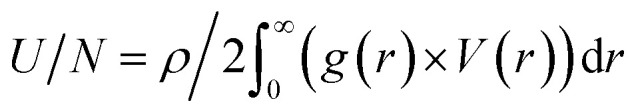
and the osmotic pressure Π of an ensemble of particles, such as:^64,65^5
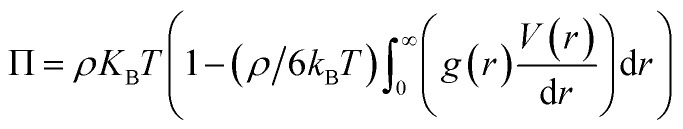


A calculation in the canonical ensemble (*N*; *V*; *T*)^65^ shows that it is also possible to calculate the isothermal compressibility using the integral over space of *g*(*r*):6
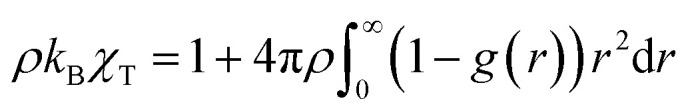


In the reciprocal space, the counterpart of the pair correlation function is the static structure factor *S*(*q*). This quantity is directly accessible by X-ray and neutron scattering experiments^66–68^ and it is defined by:7
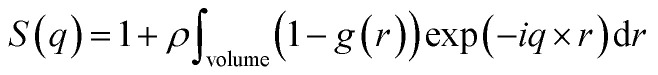


### 2.5 Molecular dynamics simulation (MD)

The MD method offers the possibility to simulate atomic and molecular systems provided that the interaction potential between the particles, that constitute the system, is known. With this method, it is possible to compare an approximate theory applied to determine the physical properties of an ensemble of particles using hypothetical interaction potential, with the experimental results. Here, we used MD simulations and OZE with the HNC closure to test the potential models representing the efficient interactions between PEO-m coated microemulsions. Generally, the flocculation of colloidal suspensions is resulting from attractive van der Waals interactions and the Brownian motion.^69,70^ Besides, kinetic stability is resulting from repulsive interactions. There are essentially two types of repulsive interactions. The first type, the repulsion results from electrostatic forces exerted between charged colloidal surfaces and which are screened by counter-ions that revolve around the microemulsion external surface. From the literature, the exact interaction potential between two charged colloids was first calculated by Derjaguin and Landau^71^ then by Verwey and Overbeek,^72^ as a part of the DLVO model. The second type consists of colloids covered by polymers. For good-solvent conditions, the adsorbed polymers represent a sufficient steric barrier, which maintains the dispersity of the colloids.

In the framework of the DLVO model, microemulsions are approximated as, neutral or charged, non-overlapping hyperspheres interacting by an efficient potential, which is the sum of a hard-sphere repulsive potential, a van der Waals attractive term and, in the case of charged microemulsions, a Yukawa type of screened coulombic interaction potential.

Besides, the addition of PEO-m on the microemulsions introduces a repellent contribution to the existing interaction potential, manifesting as interpenetration resistance between the microemulsions.^73^ In this case, the total interaction potential expressed as:

8 *V*(*r*) = *U*_HS_ + *U*_VDW_ + *U*_Coulomb_ + *V*_Steric_

where, *U*_HS_: a repulsive potential of a hard sphere is written:9
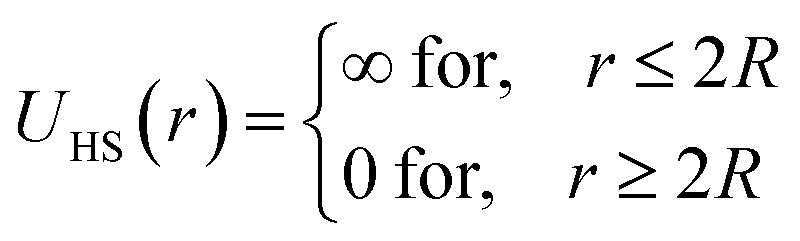
*U*_VDW_: attractive potential of van der Waals:10
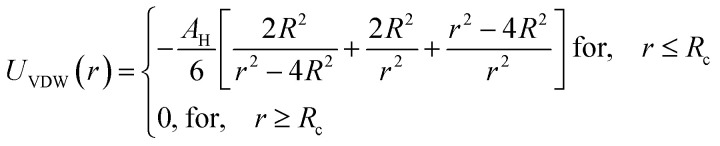
*U*_Coulomb_: a Yukawa-style coulombic repulsive potential:11
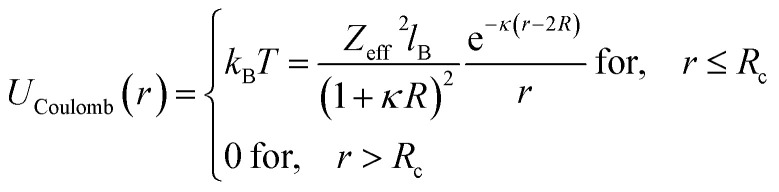
*V*_Steric_: a steric-type repulsive interaction of Yukawa-type:12
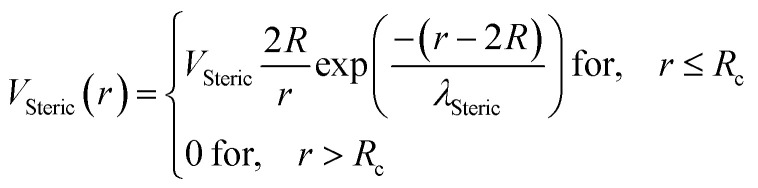
Here, *r* is the center-to-center distance, *R* = 62 Å is the microemulsion radius, *R*_c_ the cutoff distance of the pair potential, *A*_H_ = 1.1*k*_B_*T* is the Hamaker constant, *Z*_eff_ is the effective charge per nanodroplet, *κ*^−1^ represents the Debye's length, *l*_B_ is the Bjerrum length, *l*_B_ = *e*^2^/(4π*ε*_0_*ε*_r_*k*_B_*T*), with *ε*_0_ the vacuum permittivity, *ε*_r_ = 80 the dielectric constant of the solvent (water). *V*_Steric_ the potential at the contact, and *λ*_Steric_ is the range of the interaction that we assume to be of the order of the PEO-m radius of gyration. The effective-charge per microemulsion is esteemed to be *Z*_eff_ = 130 and independent to the number of added PEO-m. The dimensionless units are used to optimize the calculation time. Accordingly, the energies are reduced by unit energy *k*_B_*T*, the distance is reduced by the microemulsion radius, the time is reduced by 
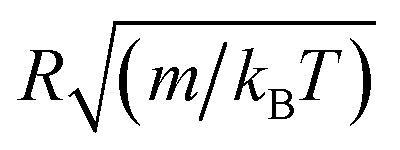
, the diffusion coefficient *D* is reduced by 
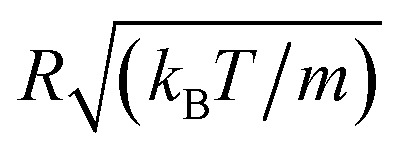
, the pressure *P* is reduced by *k*_B_*T*/*R*^3^ and the isothermal compressibility *χ*_T_ is reduced by *R*^3^/*k*_B_*T*. The MD calculations are performed for *N* = 10^6^ microemulsions enclosed in a cubic box of volume *V*, for the diluted case (*Φ* = 6.98%) and the concentrated case (*Φ* = 25.6%), with 
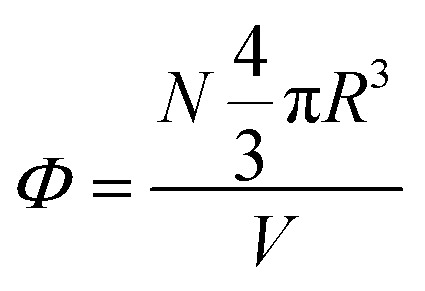
.

The equations of movement are solved in the *NVT* ensemble using the Verlet algorithm,^74,75^ with a time step δ*t* = 0.001, where the temperature is set to T* = 1. Periodic boundary conditions are applied to eliminate surface effects and simulate an infinite system.^74,75^

**Fig. 2:**
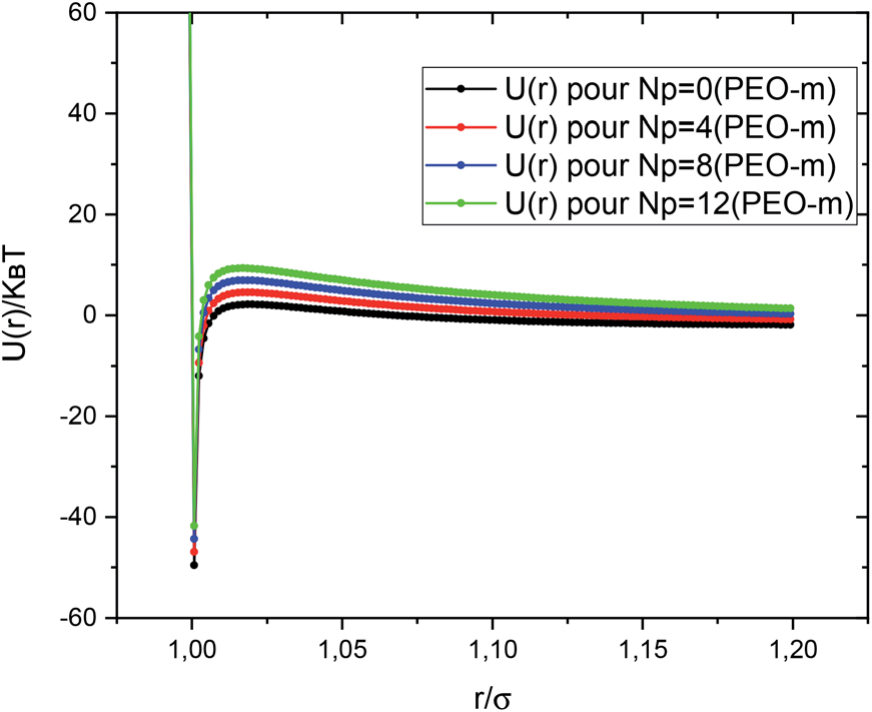
Dimensionless pair-potential between microemulsions, *U*(*r*)/*k*_B_*T*, *versus* the dimensionless distance *r*/*σ* (*σ* = 2*R*).

Fig. 2 shows the interaction potential, used in the MD simulations, plotted using the parameters discussed above, with (*V*_Steric_ = 2.6*k*_B_*T* pour *N*_p_ = 4, *V*_Steric_ = 5.2*k*_B_*T* pour *N*_p_ = 8, and *V*_Steric_ = 7.8*k*_B_*T* pour *N*_p_ = 12) and *σ* = 2*R*. We notice that the potential barrier increases proportionally to the number of added POE-m polymers per microemulsion. Thus, the repulsive forces predominate over the attractive forces. MD simulations codes are executed using the Large-scale Atomic/Molecular Massively Parallel Simulator (LAMMPS) distributed as an open-source package from Sandia National Laboratories (SNL).^76^

## 3 Results and discussions

### 3.1 Effect of PEO-m on the microemulsions shape and size

In this part, we study the effect of the added polymer PEO-m, that we note “*N*_p_”, (*N*_p_ = 4, 8, 12), on the shape and size of bare decane/water microemulsions (*N*_p_ = 0). The small-angle neutron scattering of colloidal solutions provides information on their structure.^68^ Accordingly, the microemulsions are assumed to be identical and spherical shaped, the average form factor of the polydisperse size of the colloidal suspension is:13
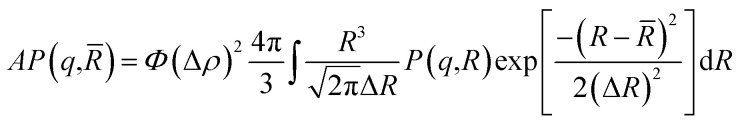
with *R̄* the mean radius and Δ*R* the standard deviation, that measures the dispersion from the individual data values to the mean radius.

**Fig. 3:**
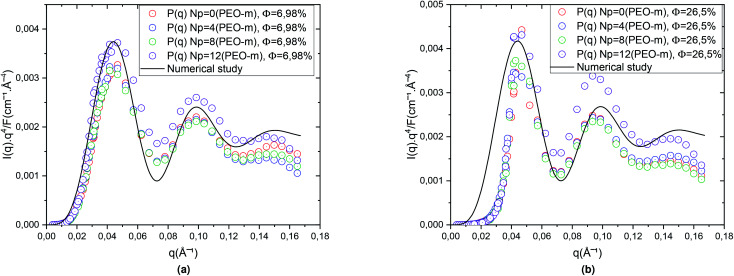
Porod representation of the scattering intensity, normalized by volume fraction *Φ*, measured for bare microemulsion (*N*_p_ = 0) and for microemulsions with three added PEO-m (*N*_p_ = 4, *N*_p_ = 8, *N*_p_ = 12). For all studied cases the numerical adjustment gives *R̄* = 62 Å, with a relative error Δ*R* = 6.2 Å. (a) Porod representation of the scattering intensity for *Φ* = 6.98%. (b) Porod representation of the scattering intensity for *Φ* = 26.5%.

In Fig. 3, the circles show the SANS spectra in the Porod representation (*q*^4^⋯*I*(*q*)), for the diluted case (*Φ* = 6.98%) and the concentrated case (*Φ* = 26.5%), for *N*_p_ = 0, 4, 8, 12. The line presents a better fit to the eqn (10) that give *R̄* = 62 Å and Δ*R* = 6.2 Å. For the diluted case (*Φ* = 6.98%), (Fig. 3a), we notice that all the spectra are superimposed independently of the parameter *N*_p_ and that the maxima and minima remain the same, for the bare microemulsion and microemulsions with different numbers of added PEO-m. Also, for the concentrated case, the volume fraction (*Φ* = 26.5%), (Fig. 3b), the spectra follow the same behaviour. While the bare microemulsions (*N*_p_ = 0) are spherical shaped, with a mean radius *R̄* = 62 Å and Δ*R* = 6.2 Å,^68^ the microemulsions are identical and well represented by spheres with a mean radius independent to the addition of different numbers of POE-m. Furthermore, we can deduce that the polymers decorate the microemulsions rather than associate with each other. These findings imply that decane/water microemulsions are identical in the presence of moderate quantities of polymers and well represented by spheres of radius *R* = 62 Å. These findings are in good agreement with a similar work studying the effect of polyethylene glycol (PEG) on the inverse microemulsion of sodium bis-(2-ethylhexyl)sulphosuccinate (AOT),^42^ and another study investigating the effect of a hydrophobically end-capped polymer (C18-PEO150-C18) on the decane/water microemulsions size.^43^

### 3.2 Effect of PEO-m on the microemulsions structural properties

In this part, we performed MD simulations using the proved interaction potential, discussed above, to study the effect of the added PEO-m per microemulsion, that we note “*N*_p_”, on *g*(*r*) and *S*(*q*) of the covered microemulsions. Thus, we considered different numbers of *N*_p_, *N*_p_ = 4, 8, and 12, and two volume-fractions, *Φ* = 6.98%, and 26.5%, corresponding, respectively, to the diluted and concentrated cases.

**Fig. 4:**
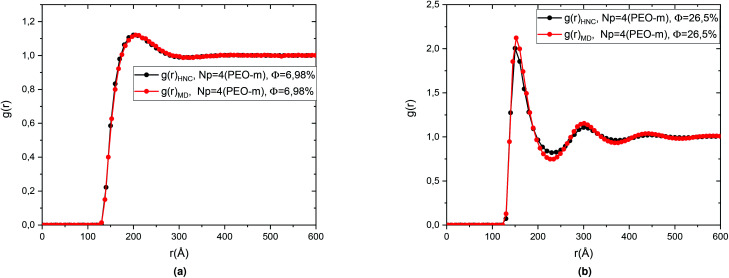
Pair correlation function *g*(*r*) of the microemulsions with four added PEO-m, obtained from the (OZ) integral equation with the closing relation (HNC) and MD simulations, for the two studied volume fractions, (a) *Φ* = 6.98% and, (b) *Φ* = 26.5%.

The Fig. 4 represents a comparison between the theoretical results of the correlation functions *g*(*r*) obtained from the numerical resolution of the integral (OZ) equation with the closure relation (HNC). And the MD simulation for microemulsion coated 4 PEO-m, in the diluted case (*Φ* = 6.98%) and the concentrated case (*Φ* = 26.5%). We note in particular that the peaks of the correlation functions from the molecular dynamics simulations are in good agreement with the corresponding values calculated from the integration equation. Also, we observe that for the concentrated case, *Φ* = 25.6%, a first peak relatively narrow found around *r* = 152.52 Å, represents the interactions between a central microemulsion and their close neighbours. Also, we notice second and third peaks, less intense and relatively large corresponding to the interactions between the second and third neighbours, respectively. For the diluted case, *Φ* = 6.98%, the correlation between the microemulsions becomes weak. Thus, the *g*(*r*) main peak height decreases regularly (from 2.123, for *Φ* = 26.5%, to 1.358, for *Φ* = 6.98%) with a displacement in the microemulsions mean-separation-distance “*r*”.

**Fig. 5:**
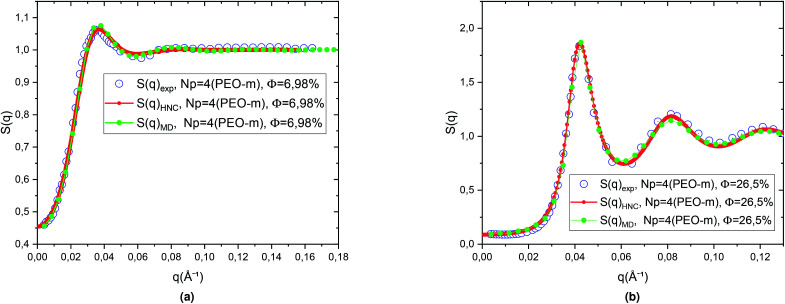
Structure factor *S*(*q*) obtained from the integral (OZ) equation with the closing relation (HNC) and MD simulations compared to SANS experiments, for microemulsions with added modified polymers PEO-m for *N*_p_ = 4 at two volume fractions, (a) *Φ* = 6.98% and, (b) *Φ* = 26.5%.

**Fig. 6:**
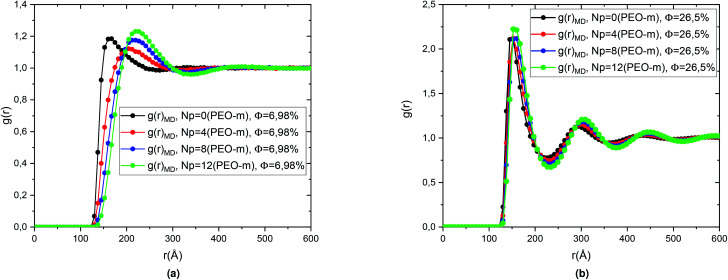
Pair correlation function *g*(*r*) obtained from MD simulations, for the microemulsions with different number of added PEO-m (*N*_p_ = 0, *N*_p_ = 4, *N*_p_ = 8, *N*_p_ = 12), for the two studied volume fractions, (a) *Φ* = 6.98% and, (b) *Φ* = 26.5%.

**Fig. 7:**
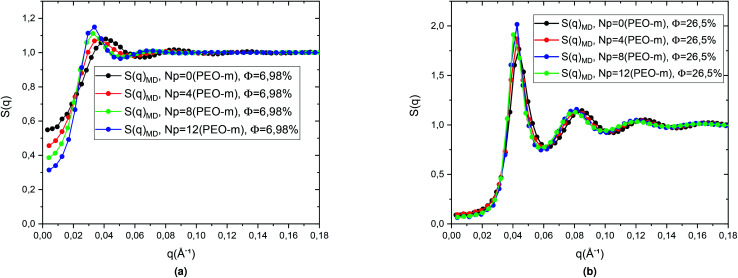
Structure factor *S*(*q*) obtained from MD simulations, for the microemulsions with different number of added PEO-m (*N*_p_ = 0, *N*_p_ = 4, *N*_p_ = 8, *N*_p_ = 12), for the two studied volume fractions (a) *Φ* = 6.98% and, (b) *Φ* = 26.5%.

We then calculated the structure factors using the Fourier transform of *g*(*r*) calculated from MD and HNC. In Fig. 5, we compare the SANS experiments findings and HNC results to those from MD simulation. We notice that the peaks of the structure factors from the two methods are almost superposed, for the two studied volume fractions. Thus, MD simulation describes well the micro-arrangement of the studied microemulsions. This observation proves the choice and the parametrization of the interaction potential used in the MD simulation. In the Fig. 6, we present the correlation function of the microemulsion covered with different number of PEO-m (*N*_p_ = 0, 4 m, 8 m, 12 m), for the volume fractions *Φ* = 6.98%, and *Φ* = 26.5%. For *Φ* = 6.98%, (Fig. 6a), we observe that the correlation peak shifts to long distances proportionally to the parameter *r*. Also, the peak becomes more pronounced, and this peak varies from (*g*_max_ = 1.18, *d* ≈ 167.4 Å) to (*g*_max_ = 1.23, *d* ≈ 219.48 Å). For *Φ* = 26.5%, (Fig. 6b), the height of the main-peak becomes more pronounced (varies from *g*_max_ = 2.12 to *g*_max_ = 2.22) with the addition of POE-m, and the position of the main-peak shifts to large distances from *d* ≈ 152.52 Å to 159.96 Å. Thus, the addition of PEO-m to the microemulsion introduces an interpenetration resistance between the microemulsions. Precisely, this repulsive effect manifests significantly for low volume fractions. In Fig. 7, we depict the structure factor *S*(*q*). In the diluted case (*Φ* = 6.98%) we notice that the principal peak becomes more intense and narrower with the increase of the parameter *N*_p_ from 0 to 12 and the position of this peak shift to small values of *q* (*q* = 2π/*d*), the same for the volume fraction, *Φ* = 26.5%, (Fig. 7b), the height of the principal peak becomes more pronounced with the addition of PEO-m and the position of the main-peak moves to small values of *q*. We notice that *S*(0), which is directly proportional to compressibility, decrease proportionally to *r*. This decrease is due to a repulsion effect between the microemulsions. Thus, these results conduct to the same conclusions according to the investigation of *S*(*q*) (Table 2).

**Table 2:** Analysis of the *S*(*q*) curves obtained by MD for the diluted case (*Φ* = 6.98%) and the concentrated case (*Φ* = 26.5%)

*r*	*Φ*	*S*(0)	*S*_max_	*d* (Å)
0	6.98%	0.55	1.08	152.6
4		0.46	1.07	165.4
8		0.39	1.11	192
12		0.31	1.14	186
0	26.5%	0.09	1.76	142.9
4		0.09	1.87	147.3
8		0.06	2.02	147.3
12		0.07	1.91	156.4

### 3.3 Effect of PEO-m polymers on the microemulsions dynamics

**Fig. 8:**
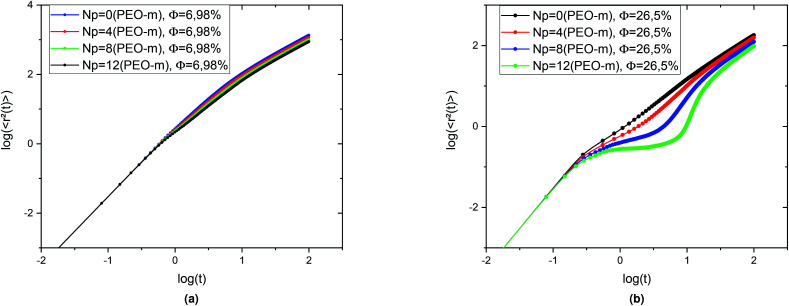
MSD as a function of time in the log–log scale, obtained from the MD simulation, for the microemulsions with different number of added PEO-m (*N*_p_ = 0, *N*_p_ = 4, *N*_p_ = 8, *N*_p_ = 12), for the two studied volume fractions, (a) *Φ* = 6.98% and, (b) *Φ* = 26.5%.

To study the influence of the addition of the PEO-m dynamics properties of the considered microemulsions, we investigate the mean-square-displacement (MSD) variation as a function of time. Fig. 8 depicts the log–log plot of the MSD as a function of time, calculated from the MD simulations for the microemulsion with added PEO-m (*N*_p_ = 0, 4, 8, 12) for the diluted case (*Φ* = 6.98%) and the concentrated case (*Φ* = 26.5%). For the diluted system, we notice that there are two regions of time-dependent, the first region at *t* < *t*_1_ = 0.665 is known as the ballistic regime, in this regime the MSDs increase linearly with time and are identical and have the same slope independently of the parameter *r*, where 
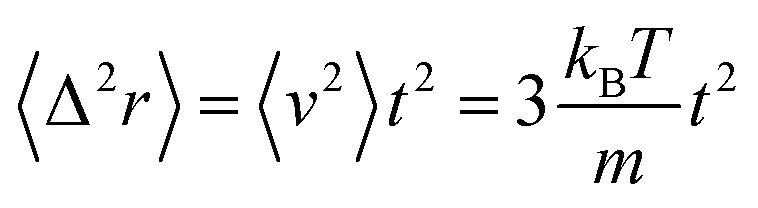
, and the second one is for longer times (*t* > *t*_1_) called the normal diffusion. In this regime, MSDs are of a linear form 〈Δ^2^*r*〉 = 6*D*_*α*=1_*t*, with *D*_*α*=1_ the normal diffusion-coefficient. This observation signifies that the random walker always undergoes normal diffusion in the diluted colloidal systems, after a short time of ballistic motion. For the dense system (*Φ* = 26.5%), an intermediate diffusion regime appears at *t* > *t*_1_ = 0.352. In this case, the MSD become sensitive to the volume fraction variation and the parameter *r*. Precisely, the MSDs show sub-diffusive behaviour, due to cage effects, this regime is called plateau regime (where 〈Δ^2^*r*〉 ∼ *t*^*α*^, (*α* < 1)), with the sub-diffusion exponent *α* naturally depends on the system parameters, namely the volume fraction and the number of hydrophobic polymers ends per microemulsion and *D* the corresponding subdiffusion coefficient. This regime implies the formation of cages, which manifest significantly as the parameter *N*_p_ increases from 0 to 12. Physically, when the number of polymers, with hydrophobic ends, per microemulsion increases, the space available for each microemulsion is reduced because of the steric repulsion. Consequently, this effect leads to a delay in the microemulsions diffusion process.

In conclusion, for the diluted case, the microemulsions diffusion is described by the usual Fickian-diffusion and the added PEO-m slow down slightly their dynamics. In contrast, for the concentrated case, the space between the microemulsions decreases. Thus, the particles trapped in cages formed by other surrounding particles. The presence of the traps makes such a diffusion process difficult. In this case, the diffusion becomes anomalous in the intermediate region (the plateau regime). The latter strongly depends on the parameter *r*.

**Fig. 9:**
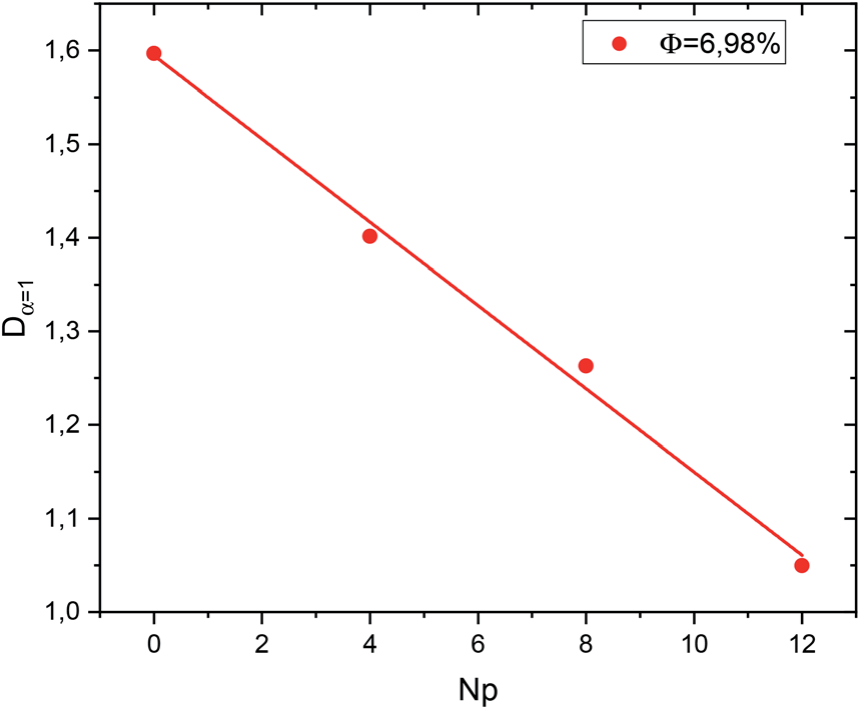
Diffusion coefficient 
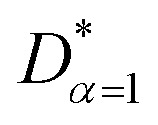
 as function of the parameter *N*_p_ for the diluted system (*Φ* = 6.98%).

In the Fig. 9 we present the normal diffusion-coefficient, *D*_*α*=1_; for the studied microemulsions as a function of the number of hydrophobic ends of the polymers by microemulsions (*r*), for the dilute system. We observe that when *N*_p_ increases, *D*_*α*=1_ decreases linearly,

14 *D*_*α*=1_ = *ar* + *D*_0_

with the slope *a* = −4.4 × 10^−2^, and *D*_*α*=10_ = 1.59 the diffusion coefficient of bare microemulsions (*N*_p_ = 0).

**Fig. 10:**
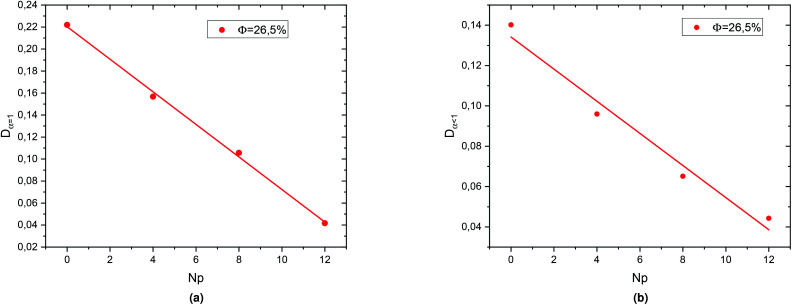
Diffusion coefficients: (a) *D*_*α*<1_ and (b) *D*_*α*=1_ as function of the parameter *N*_p_ for the dense system (*Φ* = 26.5%).

In the Fig. 10a and b we present the normal diffusion-coefficient, *D*_*α*=1_ and the sub-diffusion coefficient, *D*_*α*<1_, respectively; for the dense microemulsions system. Also, as observed for the diluted system, *D*_*α*=1_ and *D*_*α*<1_ decreases linearly as a function of the parameter *r*,

15 *D*_*α*=1_ = *ar* + *D*_(0)_

and,

16 *D*_*α*=1_ = *br* + *D*_*α*=1(0)_

with the slopes *a* = −1.48 × 10^−2^ and *b* = −7.96 × 10^−3^, and the diffusion coefficients of bare microemulsions (*N*_p_ = 0) *D*_*α*=1(0)_ = 0.22 and *D*_*α*<1(0)_ = 0.134.

This decrease in the diffusion coefficients means that the correlations between the microemulsions, which causes the cage effect, increase with the presence of hydrophobic polymers. In a recently published work, Lemaalem *et al.* studied the behaviour of the normal diffusion-coefficient of the decane/water microemulsions as a function of the volume fraction *Φ*. They noticed that when *Φ* increases, *D*_*α*=1_ decreases exponentially.^68^ Then, according to the present study, we conclude that the diffusion coefficient slowly decreases with increasing the number of added polymers, as a comparison with increasing the volume fraction. The polymer effect on the microemulsion diffusion properties found to be in good agreement with previous *in vitro* and *in silico* similar works.^43–47^

## 4 Conclusion

In this work, we study the effect of grafted PEO-dodecyl polymers (PEO-m) on the decane/water microemulsions. We combined the MD simulations method, the OZ integral equations resolved numerically using the HNC closure, and SANS experiments. We found that the structural properties obtained from the three approaches are in good agreement. SANS experiments have shown that the addition of PEO-m with different amounts on the oil/water microemulsions does not change their shape and size. Thus, the microemulsions are described by slightly polydispersed nanospheres with a mean radius of 62 Å. Analysis of the radial distribution function showed that PEO-m addition to the microemulsion induces steric-type repulsive interactions. This repulsion produces an increase in the average distance between the microemulsions and a decrease in the diffusion coefficient. Indeed, the addition of PEO-m to the oil/water microemulsion reduces the microemulsions diffusion. This decrease is significant for the dense system. But for the diluted system, this effect is slight.

We recall that the addition of PEO-m during the decane/water microemulsions preparation has several advantages in drug delivery application. Precisely in the lubrication and protection of the encapsulated drugs. Generally, the PEO-m show no acute effects on the decane/water microemulsions physicochemical properties. Thus, microemulsions covered by biocompatible and biodegradable polymers are ideal systems for transdermal drug delivery.

Finally, we underline that some polymers can increase the attraction between the microemulsions. In this case, the polymers bridge two or more microemulsions depending on their physicochemical properties. This hypothesis will be the main object of our next researches.

## Conflicts of interest

There are no conflicts to declare.

## Supplementary Material
