# Correction: Effect of hydrophobically modified PEO polymers (PEO-dodecyl) on oil/water microemulsion properties: *in vitro* and *in silico* investigations

**DOI:** 10.1039/d1ra90089g

**Published:** 2021-03-11

**Authors:** M. Khatouri, R. Ahfir, M. Lemaalem, S. El Khaoui, A. Derouiche, M. Filali

**Affiliations:** Laboratoire de Physique Appliquée, Informatique et Statistique (LPAIS), Faculty of Sciences Dhar El Mahraz, Sidi Mohamed Ben Abdellah University BP 1796 Fes Atlas Morocco; Laboratoire de Physique des Polymères et Phénomènes Critiques Sciences Faculty Ben M’Sik, Hassan II University P. O. Box 7955 Casablanca Morocco mohammedlemaalem@gmail.com

## Abstract

Correction for ‘Effect of hydrophobically modified PEO polymers (PEO-dodecyl) on oil/water microemulsion properties: *in vitro* and *in silico* investigations’ by M. Khatouri *et al.*, *RSC Adv.*, 2021, **11**, 7059–7069, DOI: 10.1039/D0RA09804C.

The authors regret that the name of one of the authors (M. Lemaalem) was shown incorrectly in the original article. The corrected author list is as shown above.

The Royal Society of Chemistry apologises for these errors and any consequent inconvenience to authors and readers.

## Supplementary Material

